# Advances in Exosomes Derived from Different Cell Sources and Cardiovascular Diseases

**DOI:** 10.1155/2020/7298687

**Published:** 2020-07-07

**Authors:** Bo Liang, Xin He, Yu-Xiu Zhao, Xiao-Xiao Zhang, Ning Gu

**Affiliations:** ^1^Nanjing University of Chinese Medicine, Nanjing, China; ^2^Hospital (T.C.M.) Affiliated to Southwest Medical University, Luzhou, China; ^3^Nanjing Hospital of Chinese Medicine Affiliated to Nanjing University of Chinese Medicine, Nanjing, China

## Abstract

Exosomes can reach distant tissues through blood circulation to communicate directly with target cells and rapidly regulate intracellular signals. Exosomes play an important role in cardiovascular pathophysiology. Different exosomes derived from different sources, and their cargos have different mechanisms of action. In addition to being biomarkers, exosomes also have a certain significance in the diagnosis, treatment, and even prevention of cardiovascular diseases. Here, we provide a review of the up-to-date applications of exosomes, derived from various sources, in the prognosis and diagnosis of cardiovascular diseases.

## 1. Introduction

Exosomes are endosomal-derived vesicles that play a critical role in cell-to-cell communication and are secreted in several biological fluids including serum, saliva, urine, ascites, and cerebrospinal fluid among others [1]. Exosomes are small (30~150 nm diameter) with a distinctive bilipid protein structure. They can carry and exchange various cargos between cells and are used as a noninvasive biomarker for several diseases. Moreover, exosomes are considered the best biomarkers for disease diagnosis, owing to their unique characteristics [2].

Recent studies have shown that the number of exosomes, exosomal encoded and noncoding RNA, and exosomal proteins may act as biomarkers for the diagnosis and prognosis of cardiovascular diseases [3], whose death brings a great threat to global health [4, 5]. Some heart protection strategies have been shown to increase the number of exosomes in the blood [6–8]. The release of exosomes in the circulation of rats with the ischemia-reperfusion model increased significantly [9], in which the main exosomes containing HSP-60 were found [10]. A group of exosomes rich extracellular vesicles purified from the blood and proved to protect rat heart and myocardial cells from acute ischemia and reperfusion injury when administered *in vivo* or *in vitro* [9]. The exosomes containing HSP70 exist in the outer membrane of normal cardiac myocytes, which can activate the MAPK/ERK1/2 signal pathway through interaction with the TLR4 receptor and thus play a role in myocardial protection [9]. Exosomes rich in miR-143 and miR-145 have atherosclerotic protective effects in mouse models [11]. In addition, the exosomes containing miR-208a are related to the level of cardiac troponin I after coronary artery bypass grafting, while miR-208a in the blood circulation does not find this relationship, which indicates that the exosomal miRNAs may be helpful for the diagnosis of cardiovascular diseases [12]. Moreover, miR-192 and miR-194 in exosomes are also considered as prognostic biomarkers of cardiovascular diseases [13]. It is worth noting that miR-34a is highly expressed in myocardial infarction and preferentially integrated into the exosomes derived from cardiomyocytes and fibroblasts [14], making it possible for early diagnosis of myocardial infarction.

**Figure 1:**
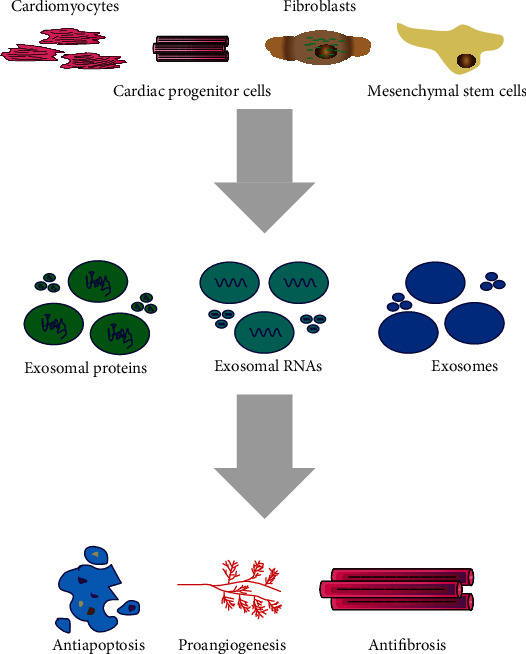
Exosomes derived from different cell sources and cardiovascular diseases.

Here, we critically review the advances in exosomes derived from different cell sources and cardiovascular diseases (Figure 1), and we mainly highlight the exosomes derived from cardiomyocytes, cardiac progenitor cells, fibroblasts, and mesenchymal stem cells, which communicate intensively to facilitate proper cardiac function through direct cell-cell contact and paracrine interactions [15, 16]. This knowledge may be helpful to promote and develop diagnostic markers and therapeutic approaches, which may be beneficial to the management of cardiovascular patients.

## 2. Exosomes Derived from Different Cell Sources

### 2.1. Exosomes Derived from Cardiomyocytes

Exosomes derived from cardiomyocytes were initially found under hypoxia and reoxygenation conditions and may contain biological molecules [17, 18]. HSPs, which play essential roles in cellular survival and adaptation under numerous stresses [19], are found to be enriched in cardiac exosomes. HSP20 contained in cardiomyocyte-derived circulating exosomes is considered to be a new type of cardiac motility factor, which can promote the formation of myocardial neovascularization by activating VEGFR2 [20] and AKT signaling pathway, repressing TNF-*α* and IL-1*β* factors to alleviate myocardial infarction [21]. HSP60, which is thought to be a danger signal to the immune system and is also highly immunogenic [22], is released via exosomes, and that within the exosome, HSP60 is tightly attached to the exosome membrane [23]. The cardiomyocyte-derived exosomes are enriched for HSP70 involved in regulating cardiomyocyte growth and survival under stress [16]. The myocyte-derived HSP90 orchestrates not only p65-mediated IL-6 synthesis but also its release in exosomal vesicles, and such exosomes and myocyte-secreted IL-6 are responsible in unison for the biphasic activation of STAT-3 signaling in cardiac fibroblasts that culminates in excess collagen synthesis, leading to severely compromised cardiac function during cardiac hypertrophy [24]. TNF-*α* can also be isolated from cardiomyocyte-derived exosomes and interact with HIF-1*α* to contribute to cardiac remodeling [25]. Furthermore, exosomes derived from cardiomyocytes were found to carry functional GLUT (GLUT4 and GLUT1) and glycolytic enzymes (lactate dehydrogenase) and were shown to have specialized functions in glucose transport and metabolism in endothelial cells [26].

Many studies have demonstrated that exosomes derived from cardiomyocytes can also carry DNA/RNA [19]. Exosomes secreted by cells derived from cardiomyocytes can improve myocardial function, inhibit cell apoptosis, and promote the proliferation of cardiomyocytes when expressed in a mouse myocardial infarction model, which may be related to the enhancement of the expression of vascular gene SIS [27]. These effects can also be achieved by increasing the expression of miR-146a, which is significantly enriched in exosomes [27]. In addition, in an adriamycin-induced dilated cardiomyopathy mouse model, systemic administration of these exosomes can reduce apoptosis and fibrosis [28]. Cardiomyocytes exert an antiangiogenic function in type 2 diabetic rats through an exosomal transfer of miR-320 into endothelial cells [29]. MiR-30a, which is derived from hypoxic cardiomyocytes, is efficiently transferred between cardiomyocytes via exosomes and regulates autophagy by affecting the expression of Beclin-1, ATG12, and the ratio of LC3II/LC3I, which are important regulators of autophagy [30]. Exosomes from cardiomyocytes were enriched for certain miRNAs (particularly miR-29b, miR-323-5p, miR-455, and miR-466) and mediated the regulation of MMP9, which is involved in matrix degradation and leads to fibrosis and myocyte uncoupling [31]. Additionally, miR-208a was upregulated in cardiomyocyte-derived exosomes and contributed to increased fibroblast proliferation and differentiation into myofibroblasts via targeting Dyrk2 [32]. In a different way, exosomes from the cardiac spheroid are used to activate fibroblasts, which can increase the secretion of angiogenic factor, SDF1 and VEGF by fibroblasts [33, 34]. When injected into the heart of chronic myocardial perfusion model rats, activated fibroblasts were found to significantly promote angiogenesis and cardiac protection [34]. Recent evidence established that exosomes secreted from cardiomyocytes can deliver a wide variety of biomolecules into other types of cells and regulate gene expression in these cells [19].

### 2.2. Exosomes Derived from Cardiac Precursor Cells

Exosomes of cardiac precursor cells can be obtained from auricular implants of patients undergoing valve surgery [35]. When these exosomes were used in myocardial infarction model rats, it was found that myocardial apoptosis decreased, angiogenesis increased, and left ventricular ejection fraction improved significantly [36]. Saphenous vein-derived pericyte progenitor cells transplantation produces a long-term improvement of cardiac function through a novel paracrine mechanism involving the secretion of miR-132 and inhibition of its target genes [37]. Microarray analysis of exosomes secreted by hypoxic cardiac precursor cells identified 11 miRNAs that were upregulated compared with exosomes secreted by cardiac precursor cells grown under normoxic conditions, and those miRNAs improved cardiac function and reduced fibrosis [38]. Cardiac precursor cell-derived exosomes have a high-level expression of GATA4-responsive-miR-451 and can protect H9C2 from oxidative stress by inhibiting caspase 3/7 activation *in vitro* and inhibit cardiomyocyte apoptosis *in vivo* [39]. Regardless of the type of stem cells used, most cells die or lose shortly after implantation. In order to solve this problem, some researchers found that cotransfection of cardiac progenitor cells and nonviral small ring plasmids carrying HIF1 can improve their resistance to hypoxia [40]; this cotransfection resulted in high expression of HIF1 in endothelial cells, production of exosomes with a high content of miR-126 and miR-210, active internalization by receptor cardiac progenitor cells, activation of kinase, and induction of glycolysis [40]. Cardiac precursor cell-derived exosomal miR-21 had an inhibiting role in the apoptosis pathway through downregulating PDCD4 [41]. Moreover, miR-21 bonded with PTEN and suppressed PTEN expressions to downregulate both infarction size and injury marker expressions *in vivo* and promote endothelial cell proliferation, inhibit apoptosis, and stimulate angiogenesis *in vitro* [42]. Human cardiac precursor cell-derived exosomes were highly enriched in miR-146a-5p and attenuated doxorubicin/trastuzumab-induced oxidative stress in cardiomyocytes through suppressing miR-146a-5p target genes Traf6, Smad4, Irak1, Nox4, and Mpo [43]. Additionally, miR-133a-cardiac progenitor cells clearly improved cardiac function in a rat myocardial infarction model by reducing fibrosis and hypertrophy and increasing vascularization and cardiomyocyte proliferation [44]. The cardiac precursor cell-secreted exosomes promote the infarct healing through the improvement of cardiomyocyte survival and angiogenesis, and the cardioprotective effects can be enhanced by hypoxia conditioning of cardiac precursor cells and are partially contributed by MALAT1 via targeting the miRNA [45]. In contrast, exosomes from cardiac progenitor cells have also been shown to stimulate cell migration [46].

### 2.3. Exosomes Derived from Fibroblasts

The results showed that the exosomes derived from fibroblasts rich in miR-21-3p could induce cardiomyocyte hypertrophy by targeting SORBS2 and PDLIM5. Inhibition of miR-21-3p reduced cardiac hypertrophy in animals treated with Ang II [47]. Besides, circulatory miR-29 and miR-30 are considered as biomarkers of left ventricular hypertrophy, the correlation of circulatory miR-21 in the diagnosis and prognosis of cardiac hypertrophy deserves further study [48]. In addition, exosomes extracted from endothelial cells expressing KLF2 can attenuate the formation of atherosclerosis [49]. It is also important that exosomes from atherosclerotic plaques and macrophages from peripheral blood participate in the development of atherosclerosis [50]. Patients with atherosclerosis have a higher level of extracellular vesicles derived from leukocyte compared with healthy participants [51].

### 2.4. Exosomes Derived from Mesenchymal Stem Cells

The exosomal proteins derived from mesenchymal stem cells can reduce the infarct area by half [52], inhibit the proliferation and migration of vascular smooth muscle [53], reduce cardiomyocyte apoptosis, promote angiogenesis, reduce ventricular remodeling, and protect cardiac function [54, 55]. Exosomes isolated from macrophage migration inhibitory factor-pretreated mesenchymal stem cells protected cardiomyocytes from apoptosis [56] through the lncRNA-NEAT1/miR-142-3p/FOXO1 signaling pathway [57]. At the same time, the exosomes derived from mesenchymal stem cells can also reduce the levels of inflammatory factors, such as IL-6 and MCP-1 [53], through activating the signal pathways involved in IGF-1/PI3K/Akt and GSK-3p [52, 54, 58, 59]. In the hypoxia-induced pulmonary hypertension mouse model, intravenous infusion of the exosomes derived from mesenchymal stem cells can also inhibit vascular remodeling and hypertension, which may be achieved by inhibiting the STAT3 signaling pathway [60]. Another interesting study confirmed the potential of mesenchymal stem cell-derived exosomes in reversing pulmonary hypertension in mice and further showed that exosomes from drug-induced pulmonary hypertension mice can induce pulmonary hypertension when injected into nondiseased animals [61]. These differences may be related to different miRNA expression patterns of exosomes.

Stem cells can be injected into the heart muscle to prevent myocardial ischemia and reperfusion injury and improve myocardial function through repair and gradual regeneration [62]. Transplantation of human CD34^+ve^ hematopoietic stem cells into ischemic tissue can induce neovascularization in preclinical models, which has been proved to be related to the treatment of angina pectoris and the improvement of exercise time in phase II clinical trials. However, the benefits in these experiments seem to depend more on the effect of paracrine signals than on stem cell transplantation of cardiomyocytes [63]. At present, exosomes have been used to regulate paracrine benefits [16, 64]. In fact, the exosomes of CD34^+ve^ have angiogenic activity *in vitro* and *in vivo*. Interestingly, although the benefits of CD34^+ve^ exosomes on angiogenesis have been observed, CD34^+ve^ hematopoietic stem cells do not have cardioprotective effects in the expression of the SHH gene after acute myocardial infarction [65]. Injection of exosomes from embryonic stem cells into the heart of mice with myocardial infarction increased neovascularization and cardiomyocyte survival and reduced fibrosis, which is related to the transmission of miR-294 and C-kit^+ve^ cardiac progenitor cells in the myocardium, thus increasing their regeneration activity [66]. In the peripheral blood of patients with chronic heart failure, CD34^+^ stem cell-derived exosomes rich in angiogenesis-related miR-126 and miR-130a were significantly reduced [67].

## 3. Future Perspectives

Exosomes act as messengers of intercellular communication among cardiomyocytes, fibroblasts, smooth muscle cells, and endothelial cells and participate in the regulation of cardiac regeneration, ventricular remodeling, and angiogenesis in cardiovascular diseases [68]. Because of its perfect properties as a carrier of signal molecules, circulating exosomes transmit both protective and harmful information [69, 70]. Since it is difficult to obtain the heart tissue samples of patients, it may be a useful strategy to detect the changes of exosomes in peripheral blood circulation to obtain the pathophysiological process information of cardiovascular diseases [8, 71] and guide the treatment of patients [61, 72]. In this new and exciting field of exosome research, there are still many problems. In contrast, there are relatively few studies on exosomal protein content and its significance in diagnosis, treatment, and prevention, although they have been proved to be accurate prognostic tools for predicting negative cardiovascular events, including CD31^+^/Annexin V^+^ and CD144-positive exosomes [73, 74]. In addition, the preparation and purification of exosomes have some limitations. Biologically, the exact mechanism of exosomes remains to be verified. The ability to study the role of exosomes *in vivo* will be greatly enhanced by the discovery of a specific production or absorption inhibitor. In terms of treatment, a better understanding of the pharmacokinetics and pharmacodynamics of exosomes is essential. All available preclinical and clinical data strongly support the hypothesis that exosomes have therapeutic value or play an important role in the treatment of many diseases [75].

The release of exosomes can also be regulated by different exogenous stimuli. Inflammatory response following myocardial infarction dramatically increased the number of circulating exosomes carrying alarmins such as IL-1*α*, IL-1*β*, TNF-*α*, and Rantes [76, 77]. In fact, recent studies demonstrated that exosome numbers may be increased by some specific chemical compounds. Monensin induces the formation of exosome and stimulates exosome secretion in K562 cells [78], which are human myeloid leukemia cells that secrete exosomes [79], in a Ca^2+^-dependent manner through an increase in transferrin [78]. Quantitative analysis demonstrates activation of histamine H1 receptor in HeLa cells which increases Ser110 phosphorylation of SNAP23, promoting multivesicular body-plasma membrane fusion and the release of CD63-enriched exosomes [80]. In addition to compounds, some drugs can also affect the secretion of exosomes. Atorvastatin enhances the numbers of exosomes derived from mesenchymal stem cells, which improve cardiac function and promote blood vessel formation, thus enhancing the therapeutic efficacy for myocardial infarction [81]. Ticagrelor, another commonly prescribed for cardiovascular diseases, can be leveraged to modulate the release of antihypoxic exosomes from resident human cardiac-derived mesenchymal progenitor cells through acute phosphorylation of ERK42/44 [82]. Besides, recent studies have shown that exosomes are also a safe alternative to drug delivery systems [83]. Ideally, a method of targeting the heart or target cells is needed to avoid intramyocardial or nonspecific systemic administration. Despite these barriers, the potential of exosomes as a therapeutic drug for cardiovascular diseases is still exciting, and there has been a blueprint for treatment based on extracellular vesicles in clinical trials [84]. However, it is important to do a lot of research on exosomes.

Exosomes can transfer proteins, RNAs, and other bioactive molecules to recipient cells to influence their biological properties [85], suggesting the potential functional diversity of exosomes [86]. The exosomes from different cardiac cells deliver a specific protein or RNA. The cardiac precursor cell-secreted exosomes rich in PAPP-A had a cardioprotection profile through releasing IGF-1 via proteolytic cleavage of IGFBP-4, resulting in IGF-1R activation and intracellular Akt and ERK1/2 phosphorylation [87]. It was found that exosomes secreted by endothelial progenitor cells derived from patients with coronary atherosclerotic heart disease could inhibit the migration and angiogenesis through expressing more miR-146a-5p and miR-146b-5p [88]. Another study indicated that normoxia endothelial progenitor cell-derived exosomes rich in miR-10b-5p could significantly ameliorate cardiac fibroblast activation *in vitro* [89].

Increasing studies also have shown that exosomes play an active role in patients with cardiovascular crisis and patients with complications. Acute myocardial infarction is a common critical disease of cardiovascular diseases. Exosomes released by chronically hypoxic cardiac precursor cells deliver a pool of miRNAs (miR-15b, miR-17, miR-20a, miR-103, miR-199a, miR-210, and miR-292) that enhance angiogenesis, reduce profibrotic gene expression, preserve myocardial contractile function, and improve cardiac function in the early hours after the onset of acute myocardial infarction [90]. Heart failure is the end-stage state of various cardiovascular diseases [91, 92], and heart failure pathological condition altered the miRNA cargos of cardiac-derived exosomes and impaired their regenerative activities. It may be related to miR-21-5p, the exosome released from explant-derived cardiac stromal cells, contributing to heart repair by enhancing angiogenesis and cardiomyocyte survival through the phosphatase and tensin homolog/Akt pathway [93]. Both heart failure and diabetes independently increase the morbidity of another disease and associated with considerable mortality [94, 95]. Diabetic cardiomyocytes exhibited increased secretion of detrimental exosomes containing decreased HSP20 levels, which contributed to diabetes-induced organ damage [96]. In a transgenic mouse model with cardiac-specific overexpression of HSP20, overexpression of HSP20 significantly attenuated cardiac dysfunction, hypertrophy, apoptosis, fibrosis, and microvascular rarefaction, in other words, protected against *in vivo* cardiac adverse remodeling by increasing generation/secretion of exosomes, indicating that elevation of HSP20 in cardiomyocytes can offer protection in diabetic hearts through the release of instrumental exosomes [96]. Poststroke cardiac complications are common, and diabetes exacerbates poststroke cardiac injury. In type 2 diabetes mellitus-stroke mice, exosomes harvested from human umbilical cord blood-derived CD133^+^ cell treatment significantly increased miR-126 expression in the heart and decreased its target gene expression and decreased myocardial cross-sectional area, interstitial fibrosis, TGF*β*, numbers of M1 macrophages, and oxidative stress markers 4-HNE and NOX2 in heart tissue, meaning that exosome treatment significantly improves cardiac function [97]. Additionally, uremic cardiomyopathy contributes to chronic kidney disease-induced morbidity and mortality. Overexpression of exosome-encapsulated miR-26a in muscle attenuated cardiomyopathy via exosome-mediated miR-26a transfer, which may be related to decreased the upregulation of FBXO32/atrogin-1 and TRIM63/MuRF1 and depressed cardiac fibrosis lesions [98]. Although exosomes show exciting and gratifying cardioprotection effects, it should be noted that the evidences of this cardioprotection are mostly from *in vivo* or *in vitro*, which still needs to be verified in the clinic.

## 4. Conclusion

In this review, ample preclinical and biomedical data were summarized (Table 1), which can provide a reference for the study of exosomes and their application in the diagnosis and prognosis of cardiovascular diseases. Although the current evidences show that exosomes derived from different cell sources are helpful for the diagnosis and prognosis of cardiovascular diseases, most of the evidences come from preclinical studies; solid clinical data are urgently needed. Moreover, there are some limitations in the preparation and purification of exosomes, and their exact mechanisms of action still need to be validated. These challenges need to be addressed before exosomes can proceed to clinical application.

## Figures and Tables

**Table 1:** Summary of exosomes derived from different cell sources in cardiovascular diseases.

Source	Cargos	Biological effects	Evidences	References
Cardiomyocytes	HSP20	Promote angiogenesis by activating VEGFR2, and activate AKT signaling pathway and repress TNF-*α* and IL-1*β* factors to alleviate myocardial infarction	Preclinical evidences (*in vivo* and *in vitro*)	[20, 21]
	HSP60	Promote immune responses	Preclinical evidences (*in vitro*)	[23]
	HSP70	Activate monocytes alone, resulting in monocyte adhesion to endothelial cells; improve cardiac function	Preclinical evidences (*in vitro*)	[16, 17]
	HSP90 and IL-6	Active STAT-3 signaling in cardiac fibroblasts that culminates in excess collagen synthesis, leading to severely compromised cardiac function during cardiac hypertrophy	Preclinical evidences (*in vivo* and *in vitro*)	[24]
	TNF-*α*	Interact with HIF-1*α* to contribute to cardiac remodeling	Preclinical evidences (*in vitro*)	[25]
	GLUT	Increase glucose transport	Preclinical evidences (*in vitro*)	[26]
	miR-15b, miR-17, miR-20a, miR-103, miR-199a, miR-210, and miR-292	Enhance angiogenesis, reduce profibrotic gene expression, preserve myocardial contractile function, and improve cardiac function	Preclinical evidences (*in vivo* and *in vitro*)	[90]
	miR-29b, miR-323-5p, miR-455, and miR-466	Mediate the regulation of MMP9, which is involved in matrix degradation and leads to fibrosis and myocyte uncoupling	Preclinical evidences (*in vitro*)	[31]
	miR-30a	Regulate autophagy by affecting the expression of Beclin-1, ATG12, and the ratio of LC3II/LC3I	Preclinical evidences (*in vitro*)	[30]
	miR-34a	Biomarkers of myocardial infarction	Preclinical evidences (*in vitro*)	[14]
	miR-146a	Inhibit apoptosis and promote proliferation of cardiomyocytes, while enhancing angiogenesis	Preclinical evidences (*in vivo* and *in vitro*)	[27]
	miR-208a	Increase fibroblast proliferation and differentiation into myofibroblasts via targeting Dyrk2	Preclinical evidences (*in vivo* and *in vitro*)	[32]
	miR-320	Inhibit proliferation, migration, and tube-like formation	Preclinical evidences (*in vivo* and *in vitro*)	[29]
	miR-451	Protect H9C2 from oxidative stress by inhibiting caspase 3/7 activation and inhibit cardiomyocyte apoptosis	Preclinical evidences (*in vivo* and *in vitro*)	[39]
	NA	Reduce apoptosis and fibrosis	Preclinical evidences (*in vivo* and *in vitro*)	[28]
	NA	Activate fibroblasts, which can increase the secretion of angiogenic factor, SDF1 and VEGF by fibroblasts	Preclinical evidences (*in vivo* and *in vitro*)	[33, 34]
	NA	Promote angiogenesis and cardiac protection	Preclinical evidences (*in vivo* and *in vitro*)	[34]

Cardiac progenitor cells	PAPP-A	Cardioprotection profile through releasing IGF-1 via proteolytic cleavage of IGFBP-4, resulting in IGF-1R activation, intracellular Akt and ERK1/2 phosphorylation	Preclinical evidences (*in vivo* and *in vitro*)	[87]
	miR-15b and miR-20a	Stimulate angiogenesis	Preclinical evidences (*in vivo* and *in vitro*)	[38]
	miR-17 and miR-103	Promote angiogenesis, inhibit myocardial fibrosis	Preclinical evidences (*in vivo* and *in vitro*)	[38]
	miR-21	Inhibit cardiomyocyte apoptosis through downregulating PDCD4; downregulate both infarction size and injury marker expressions *in vivo* and promote endothelial cell proliferation, inhibit the apoptosis, and stimulate angiogenesis *in vitro* by targeting PTEN	Preclinical evidences (*in vivo* and *in vitro*)	[41, 42]
	miR-126 and miR-210	Active kinase and induce glycolysis	Preclinical evidences (*in vivo* and *in vitro*)	[40]
	miR-132, miR-210, and miR-146a-3p	Decrease myocardial apoptosis, increase angiogenesis, and improve left ventricular ejection fraction	Preclinical evidences (*in vivo* and *in vitro*)	[27, 36, 37]
	miR-133a	Improve cardiac function by reducing fibrosis and hypertrophy and increasing vascularization and cardiomyocyte proliferation	Preclinical evidences (*in vivo* and *in vitro*)	[44]
	miR-146a-5p	Attenuate doxorubicin/trastuzumab-induced oxidative stress in cardiomyocytes through suppressing target genes Traf6, Smad4, Irak1, Nox4, and Mpo	Clinical evidences	[43]
	miR-181a and miR-323-5p	Promote angiogenesis	Preclinical evidences (*in vivo* and *in vitro*)	[27, 36]
	miR-210	Promote angiogenesis, inhibit cardiomyocyte apoptosis, improve heart function	Preclinical evidences (*in vivo* and *in vitro*)	[38]
	lnc RNA MALAT1	Promote the infarct healing through improvement of cardiomyocyte survival and angiogenesis by targeting the miRNA	Preclinical evidences (*in vivo* and *in vitro*)	[45]
	NA	Stimulate cell migration	Preclinical evidences (*in vitro*)	[46]

Fibroblasts	miR-21-3p	Induce cardiomyocyte hypertrophy by targeting SORBS2 and PDLIM5	Preclinical evidences (*in vivo* and *in vitro*)	[47]
	miR-21, miR-29, and miR-30	Biomarkers of left ventricular hypertrophy	Preclinical evidences (*in vivo* and *in vitro*)	[48]
	miR-34a	Biomarkers of myocardial infarction	Preclinical evidences (*in vitro*)	[14]

Mesenchymal stem cells	NA	Reduce the infarct area, inhibit the proliferation and migration of vascular smooth muscle, reduce cardiomyocyte apoptosis, promote angiogenesis, reduce ventricular remodeling, and protect cardiac function	Preclinical evidences (*in vivo* and *in vitro*)	[52–55]
	NA	Protect cardiomyocytes from apoptosis through lncRNA-NEAT1/miR-142-3p/FOXO1 signaling pathway	Preclinical evidences (*in vitro*)	[56, 57]
	NA	Reduce the levels of inflammatory factors, such as IL-6 and MCP-1, through activating the signal pathways involved in IGF-1/PI3K/Akt and GSK-3p	Preclinical evidences (*in vivo* and *in vitro*)	[52–54, 58, 59]
	NA	Inhibit vascular remodeling and hypertension by inhibiting STAT3 signaling pathway	Preclinical evidences (*in vivo* and *in vitro*)	[60]
	NA	Reverse pulmonary hypertension	Preclinical evidences (*in vivo*)	[61]
	miR-126 and miR-130a	Biomarkers of chronic heart failure	Clinical evidences	[67]
	miR-294	Increase neovascularization, cardiomyocyte survival, and reduce fibrosis	Preclinical evidences (*in vivo* and *in vitro*)	[66]

Endothelial cells	KLF2	Attenuate the formation of atherosclerosis	Preclinical evidences (*in vivo* and *in vitro*)	[49]
	miR-10b-5p	Ameliorate cardiac fibroblast activation	Preclinical evidences (*in vitro*)	[89]
	miR-146a-5p and miR-146b-5p	Inhibit the migration and angiogenesis	Preclinical evidences (*in vitro*)	[88]

Macrophages and leukocyte	NA	Promote vascular smooth muscle cells migration and adhesion, which may be mediated by the integration of extracellular vesicles into vascular smooth muscle cells and the subsequent downstream activation of ERK and Akt	Preclinical evidences (*in vitro*)	[51]
		Biomarkers of atherosclerosis	Clinical evidences	[50]
Cardiac stromal cells	miR-21-5p	Contribute to heart repair by enhancing angiogenesis and cardiomyocyte survival through the phosphatase and tensin homolog/Akt pathway	Pre-clinical evidences (*in vivo* and *in vitro*)	[93]
